# Hand Injuries by the Killer Kite Manja and Their Management

**Published:** 2017-05

**Authors:** Mohd Altaf Mir, Adil Mahmud Ali, Mohd Yaseen, Arshad Hafeez Khan

**Affiliations:** Department of Burns, Plastic and Reconstructive Surgery, Jawaharlal Nehru Medical College, AMU, Aligarh, India

**Keywords:** Kite string, Hand injury, Tendon, Nerve, Digit

## Abstract

Although hand injuries due to kite strings seem to be trivial, these injuries could be serious enough to lose the function of hand. This case series in the division of Plastic and Reconstructive Surgery of our institution from August 2014 to January 2016 evaluated the clinic-etiological profile, severity and management of hand injuries due to kite strings assessed clinically and radiologically. Eleven patients reported kite related injuries during two years, and 5 presented during 17 months. Of 11 patients, 8 were male and 3 were female with a M:F ratio of 2.66:1. The majority of patients presented with the mean age of 19.9±4.27 years. Eight patients presented acutely to the emergency while 3 believed the injury to be trivial and had delayed presentation. Injuries in the right hand were 8 and 3 in the left hand. Seven patients had injuries in zone II of the hand while 4 presented with zone III injuries. Total number of injured digits was 14 (1.4±1.11), total number of injured tendons was 26 (2.36±2.18), only one patient had nerve injury (mean=0.09), and no patient had any major vessel injury. So strict attention to safety measures and parental/guardian supervision while flying kites can avoid many preventable injuries to life and limb and also let the sport be an enjoyable and safe.

## INTRODUCTION

Every sport or game has certain injuries unique to it, even a seemingly harmless and docile sport like flying a kite. Kites were first introduced by the Chinese more than three thousand years ago^1^ and were flown to bring good luck, they are flown throughout the world as a leisure pass time, but in places like India, Pakistan and Afghanistan, Kite flying is more like a battle of which opponent can bring down the others kite and claim the prize!^1^

**Fig. 1:**
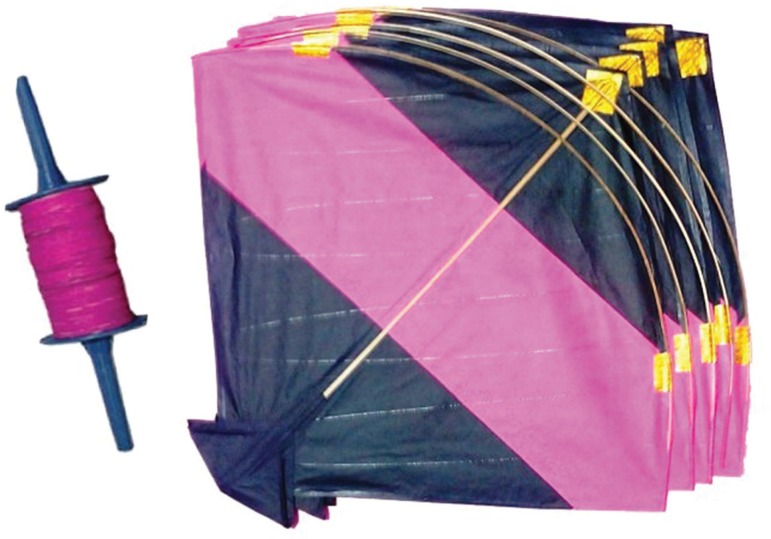
Kite and Manja (string

Injuries of kite flying activity are commonly sustained by kite-flyers, kite-runners, riders of two wheelers and pedestrians. Injuries related to flying kites can be indirect like falls from height while flying kites to direct injuries by “the Killer Kite Manja”. “Manja” is the fine string attached to the kite (Figure 1) and it is made more lethal by coating it with powdered glass or other chemicals, so that one can cut down the opponents kite more easily. The “Manja” being usually white in color and very fine is not easily visible and many a stray kite “Manjas” have been reported to cause a wide spectrum of minor to severe injuries including deaths.^2^

Reports of electrocution through a copper string attached to the kite in children have also been reported.^3^^,^^4^ The distribution of Kite related injuries varies over different locations and regions and is influenced by the local cultures and traditions, like there is an epidemic of kite related injuries during the festival of Makar Sankranti in the states of Gujrat and Rajasthan in January or the Basant festival in Peshawar, Pakistan. While a few articles do speak about the spectrum of kite related injuries, exclusive literature focusing on hand injuries related to the kite “Manja” is seldom found.^3^^,^^4^

We present a prospective observational study over a period of two years from 2014 to 2015 dealing with exclusive Hand injuries related to the kite “Manja” that presented to the Post Graduate Department of Reconstructive and Plastic Surgery at our institute and our subsequent management. Patients of all ages and sex with Hand Injuries exclusively related to flying kites were included in this prospective study. The study was conducted over a period of 17 months from August 2014 to January 2016. The patients with hand injuries due to kite string were admitted in our emergency division of plastic and reconstructive surgery. Injuries of hand were assessed clinically and radiologically. Each wound was explored in emergency operation room for the final assessment and definitive management of the injuries. Patient profile and mode of injury; number of digits and tendons involved, any nerve or major vessel damage and our subsequent management was documented and is summarized in Table 1 and 2.

**Table 1:** Clinical summary of cases

**Case No**	**Age** **(years)**	**Sex**	**Presentation **	**Zones of hand injury**	**No of digits injured**	**No of tendons injured**	**No of nerves injured**	**No of vessels injured**	**Management**	**Complication **
1	18	Male	Delayed	Zone II left hand	2	4 (FDS and FDP)	No	No	Tenorrhaphy	No
2(fig 2 and 3)	16	Male	Acute	Zone II right hand thumb	1	1( FPL)	No	No	Tenorrhaphy	No
3(fig 4)	22	Male	Acute	Zone III left hand laceration alone	0	0	No	No	Primary repair	No
4	28	Male	Delayed	Zone III right hand	1	2(FDS and FDP)	No	No	Tenorrhaphy	No
5	20	Male	Acute	Zone III right hand laceration alone	0	0	No	No	Primary repair	No
6	28	Male	Delayed	Zone II right hand	2	4 (FDS and FDP)	Lateral branch of median nerve	No	Tenorrhaphy, epineural neurorrhaphy	No
7	15	Female	Acute	Zone II right hand	3	6(FDS and FDP)	No	No	Tenorrhaphy	No
8(fig 5 and 6)	16	Female	Acute	Zone II left hand	3	5(FDS and FDP)	No	No	Tenorrhaphy	No
9	18	Female	Acute	Zone II right hand laceration alone	0	0	No	No	Primary repair	No
10	18	Male	Acute	Zone II right hand	2	4(FDS and FDP)	No	No	Tenorrhaphy	No
11	22	Male	Acute	Zone III right hand laceration alone	0	0	No	No	Primary repair	No

FDS=Flexor digitorum superficialis, FDP=Flexor digitorum profundus, FPL=Flexor policis longus

**Table 2:** Management of hand injuries due to kite strings

**Management **	**Number of patients**	**Number of digits **	**Number of tendons **	**Number of nerves **	**Number of vessels**
Primary closure of wound alone	4	-	-	-	-
Tennorrhaphy	7	14	26	-	-
Neurrhorrhaphy	1	1	-	1	-
Revascularization	0	0	-	-	0
Total	11	14(1.4±1.11)	26(2.36±2.18)	1(0.09)	0

**Fig. 2:**
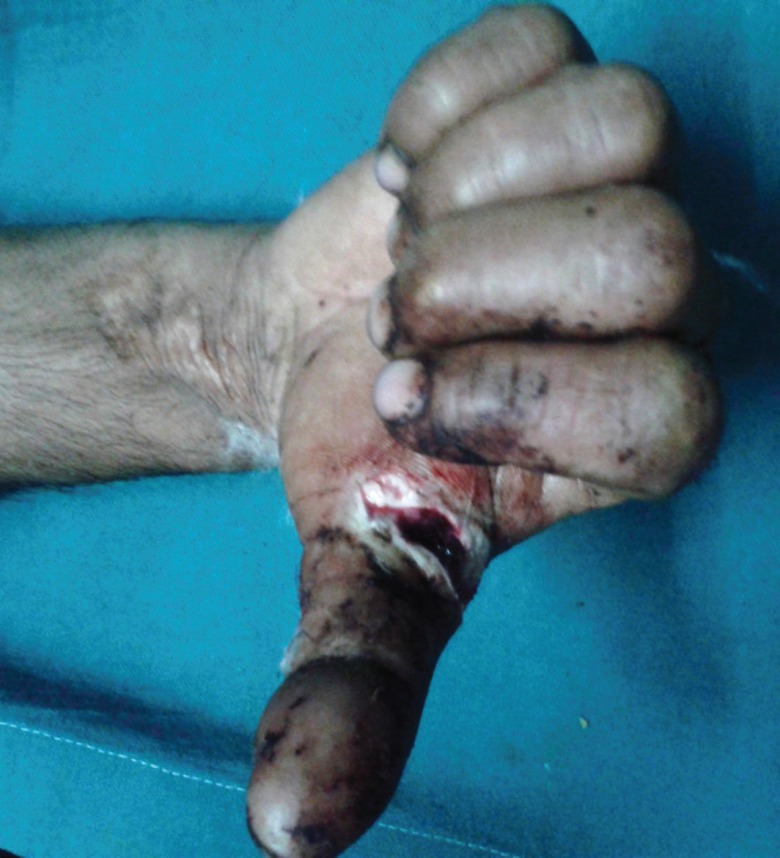
Preoperative photograph in case 2

**Fig. 3:**
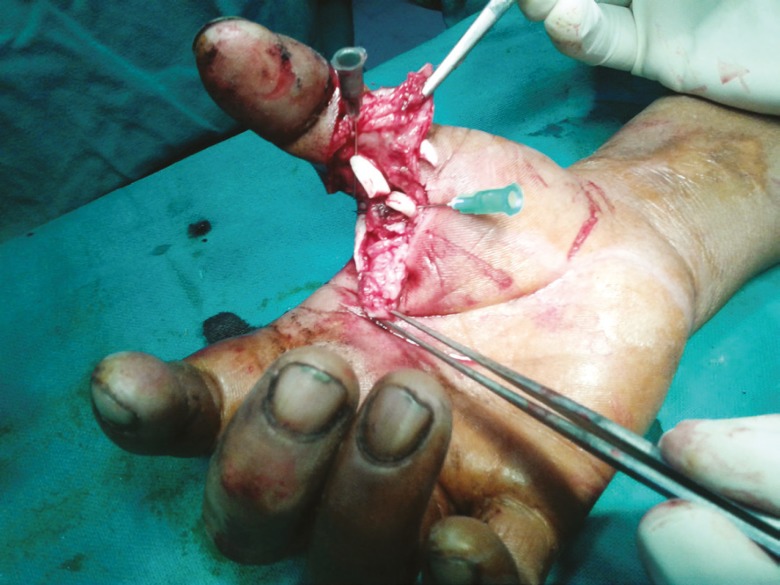
Intraoperative photograph in case 2 depicting FPL injury.

**Fig. 4:**
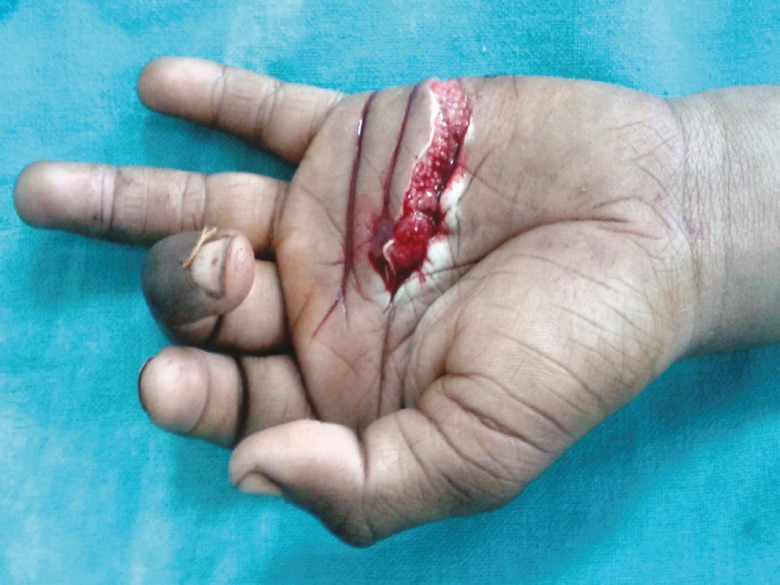
Lacerated wound in case 3.

**Fig. 5:**
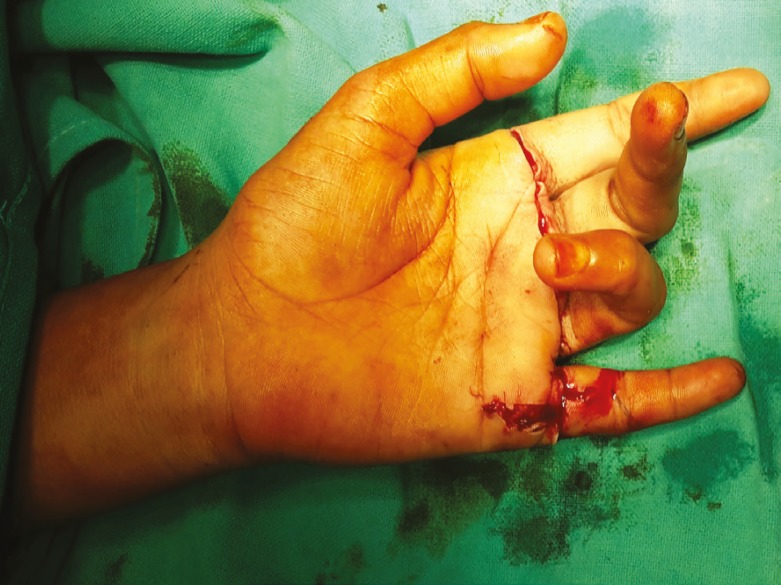
Preoperative photograph case in 8.

**Fig. 6:**
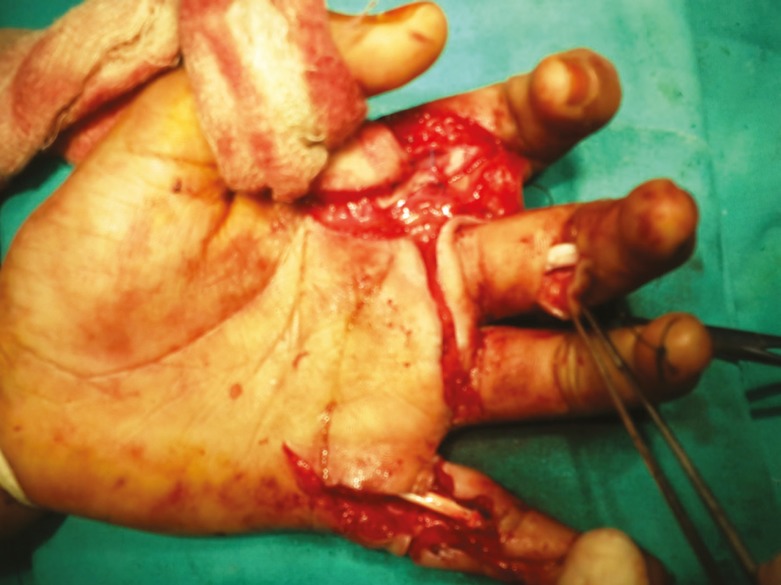
Intraoperative photograph in case 8 depicting multiple flexor tendons injured.

A total of 11 patients reported kite related injuries to our institute over a period of two years, 5 patients presented over a period of 17 months from August 2014 to January 2016. Of the 11 patients 8 were male and 3 were female with a M:F ratio of 2.66:1. The majority of patients presented in the younger age group with the mean age of presentation of 19.9±4.27 years. Majority of patients (8) presented acutely to the emergency while a few (3) patients thinking the injury to be trivial had delayed presentation. Patients who presented with injuries to the right hand were 8 while 3 presented with injuries to the left hand. Majority of patients (7) had injuries in zone II of the hand while 4 patients presented with zone III injuries. Total number of digits injured were 14 (1.4±1.11), total number of tendons injured were 26(2.36±2.18), only one patient had nerve injury (mean=0.09), no patient had any major vessel injury in our study (Table 1 and 2, Figure 2-6).

## DISCUSSION

Kite flying is a popular leisure activity throughout the world. Certain regions like Gujrat and Rajasthan in India glorify the Sun God by have kiting festivals in the month of January (Makar Sankranti) or places like Peshawar in Pakistan celebrate the coming of spring (Basant), these being the more well known of the organized kiting competitions. Injuries related to kite flying can be indirectly associated like falls from height while flying kites with head injuries or fractures. In the study conducted by Mehmood *et al.* in Peshawar in 2009, 139 patients with kite related injuries were admitted, they found simple lacerations and cuts to fingers to be the most common presentation (29%) followed by bruises (21%) neck injuries were seen in 15 (11%) patients. The study also found the maximum injuries (63%) occurred in age group of 10-29 years with a male: female ratio of 9:1.^5^

The “Manja” or thread attached to the kite is the major cause of direct kite injuries its edges being sharpened by ground glass. The loose thread may get entangled to the neck of a high speed motorist and cause serious cut throat like injuries. Singh *et al.* reported a serious maxillofacial laceration in a child sitting in front of a motorcycle when a stray kite manja cut him.^6^ Kite flying being a popular sport in regions of Brazil also, Ventura *et al.* have reported 13 patients with cervical injuries in their study^7^ with zone II and zone III injuries neck, a case report from Babu et al also reported a zone II cervical region injury to a motorcyclist with injury to the external jugular vein being the most commonly reported.^8^

Not much published literature could be found on types of hand injuries related to flying kites. Of the reported literature hand injuries to the palmer surface of the index finger and thumb of the dominant side predominate^3^ as is also true with our study. Kite string injuries are not only fatal to humans but birds too. Gujrat, India, is an important breeding ground for the Critically Endangered White-rumped Vulture Gyps bengalensis.^9^ Every year in the month of January during the Makar Sankranti festival a lot of birds like pigeons, crows etc including these endangered vultures are injured or have suffered serious injuries to life and wing. A rescue mission conducted from January 2009 and August 2012 showed that of the 108 vultures rescued kite related injuries were 43.9% of cases.^9^

The study published by Gupta et al also showed the wide spectrum of cases related to kite string injuries during the Makar Sankranti festival in Gujrat, India.^10^ Kite string injuries though not usually thought so has been the cause of deaths in many patients.^11^ Kite flying and “kite wars” are a popular sport usually among the adolescent and young age group and most of the patients in our study too were of the mean age of 19.90±4.27 years. As in any mode of injury prevention is always better than cure, strict attention to safety measures and parental/guardian supervision can avoid many preventable injuries to life and limb and also let the sport be an enjoyable and safe one.^12^

Nadir Mehmood *et al.*^5^ in their study mentioned falls and head injuries while flying kites to be the most important cause of morbidities. Falls while flying kites are a leading cause of the global burden of injury to children^13^^,^^14^ resulting in more than 37,000 deaths annually for those aged 15 years.^15^ As in any mode of injury prevention is always better than cure, strict attention to safety measures and parental/guardian supervision while flying kites can avoid many preventable injuries *to* life and limb and also let the sport be an enjoyable and safe.

## CONFLICT OF INTEREST

The authors declare no conflict of interest.
